# The Limbic Degradation of Aging Brain: A Quantitative Analysis with Diffusion Tensor Imaging

**DOI:** 10.1155/2014/196513

**Published:** 2014-04-13

**Authors:** Hediye Pınar Gunbey, Karabekir Ercan, Ayşe Serap Fındıkoglu, H. Taner Bulut, Mustafa Karaoglanoglu, Halil Arslan

**Affiliations:** ^1^Radiology Department, Ondokuz Mayıs University, Kurupelit, 55139 Samsun, Turkey; ^2^Radiology Department, Ataturk Research and Education Hospital, Ankara, Turkey; ^3^Radiology Department, Medipol University Hospital, Istanbul, Turkey; ^4^Radiology Department, Adıyaman University, Adıyaman, Turkey

## Abstract

*Introduction*. The limbic system primarily responsible for our emotional life and memories is known to undergo degradation with aging and diffusion tensor imaging (DTI) is capable of revealing the white matter integrity. The aim of this study is to investigate age-related changes of quantitative diffusivity parameters and fiber characteristics on limbic system in healthy volunteers. *Methods*. 31 healthy subjects aged 25–70 years were examined at 1,5 TMR. Quantitative fiber tracking was performed of fornix, cingulum, and the parahippocampal gyrus. The fractional anisotropy (FA) and apparent diffusion coefficient (ADC) measurements of bilateral hippocampus, amygdala, fornix, cingulum, and parahippocampal gyrus were obtained as related components. *Results*. The FA values of left hippocampus, bilateral parahippocampal gyrus, and fornix showed negative correlations with aging. The ADC values of right amygdala and left cingulum interestingly showed negative relation and the left hippocampus represented positive relation with age. The cingulum showed no correlation. The significant relative changes per decade of age were found in the cingulum and parahippocampal gyrus FA measurements. *Conclusion*. Our approach shows that aging affects hippocampus, parahippocampus, and fornix significantly but not cingulum. These findings reveal age-related changes of limbic system in normal population that may contribute to future DTI studies.

## 1. Introduction

The limbic system is a group of interconnected structures that mediate emotions, learning, and memory. It directly connects the lower and higher brain functions and influences emotions, the visceral responses to those emotions, motivation, mood, and sensations of pain and pleasure. It is composed of a group of interconnected gray and white matter structures that create a loop in each cerebral hemisphere. Papez in 1937 described the set of connections in the limbic system that link the hippocampus, mamillary bodies, thalamus, cingulated, and parahippocampal gyrus [1]. The fornix and the cingulum, the most visible white matter connections of this circuit, connect cortical and subcortical brain structures. The fornix projects from the hippocampal formation to the hypothalamus, while the cingulum connects the cingulate and the parahippocampal gyri to the septal cortex. Both the gray and white matter components of the limbic system have been studied using MR imaging in several brain disorders, such as epilepsy [2–4], dementia [5], and schizophrenia [6]. With normal aging, many volumetric studies reported a reduction in hippocampal, parahippocampal, and cingulated volumes [7–9] while others did not find evidence for age-related volume losses in these structures [10, 11]. Fiber tracking studies focused on limbic connections [12] and potential changes with aging [13] revealed a new viewpoint for this interesting structure of the human brain. However, to the best of our knowledge none of these studies investigated the integrity of whole components, including the hippocampus, amygdala, parahippocampal gyrus, cingulum, and fornix together with diffusion tensor imaging (DTI).

DTI is a noninvasive specific neuroimaging technique that enables measurement of restricted water diffusion in brain tissue. It is a more sensitive imaging method than qualitative observation for investigating white matter structures. It has revealed evidence of microstructural disruption of brain white matter in healthy adults as they age, even in regions appearing normal on conventional volume imaging [14]. The two principal DTI metrics are fractional anisotropy (FA), which represents the directionality and mean diffusivity (MD), and the magnitude of water diffusion [15]. The FA as an indicator of white matter coherence and axonal organization may be influenced by myelination, orientation, coherence, packing density, and structural integrity of neural fiber tracts. Highly myelinated fiber bundles with a common orientation will have high anisotropy and disruption of the myelin sheath, such as with aging, and can result in increase in extracellular water content and mean diffusivity [16]. Axonal damage has been correlated with decreased FA and increased MD [17]. The architecture of white matter, which restricts water movement perpendicular to the fiber axis, is especially suitable for DTI analysis, as it allows three-dimensional (3-D) characterization of fiber tracts and comparison of white matter structures between populations.

In cooperation with DTI and tractography, the aim of this study is to characterize the microstructural effects of aging on the limbic system interconnected components and relationship between them in course of time. Investigating the limbic system including several components in a broad perspective may be more informative in understanding the effects of aging on emotions, memory, attention, and social processing.

## 2. Materials and Methods

### 2.1. Subjects

This retrospective study included thirty-one subjects with no self-reported history of neurological or psychiatric disease or brain injury, aged 25–70 years (mean ± standard deviation: 49.39 ± 14.94, 17 males, 14 females). Subjects were approximately equally distributed across the age range (Table 1). The subjects were recruited from our data base. To exclude dementia the elder 14 subjects ≥50 years old who have Mini Mental State Examination (MMSE) score evaluated by physician were included in the study. The inclusion criterion for minimum MMSE score was chosen 27 points with similar other aging studies [18–20].

### 2.2. MR Imaging Protocol

All subjects were scanned on a 1.5 TMR scanner (Philips Achieva, The Netherlands). Slew rate 40 mT/m) with an eight-channel head coil. A standard conventional MR imaging protocol included axial and sagittal T2-weighted turbo spin-echo (TSE) (TR/TE = 5000/100 ms, slice thickness (thk): 5 mm), axial fluid-attenuated inversion recovery (FLAIR) (TR/TE = 6000/120 ms, IR: 2000 ms, thk: 2 mm) sequence, and a T1-weighted 3D magnetization prepared rapid acquisition gradient-echo sequence (MPRAGE) (TR = 7.2/TE = 3,2 ms, NSA = 1, FOV = 256 mm, slice thk: 1 mm, gap = 0 mm, flip angle = 8°, matrix = 256 × 256 pixels) through 160 slices of the entire brain.

**Figure 1:**
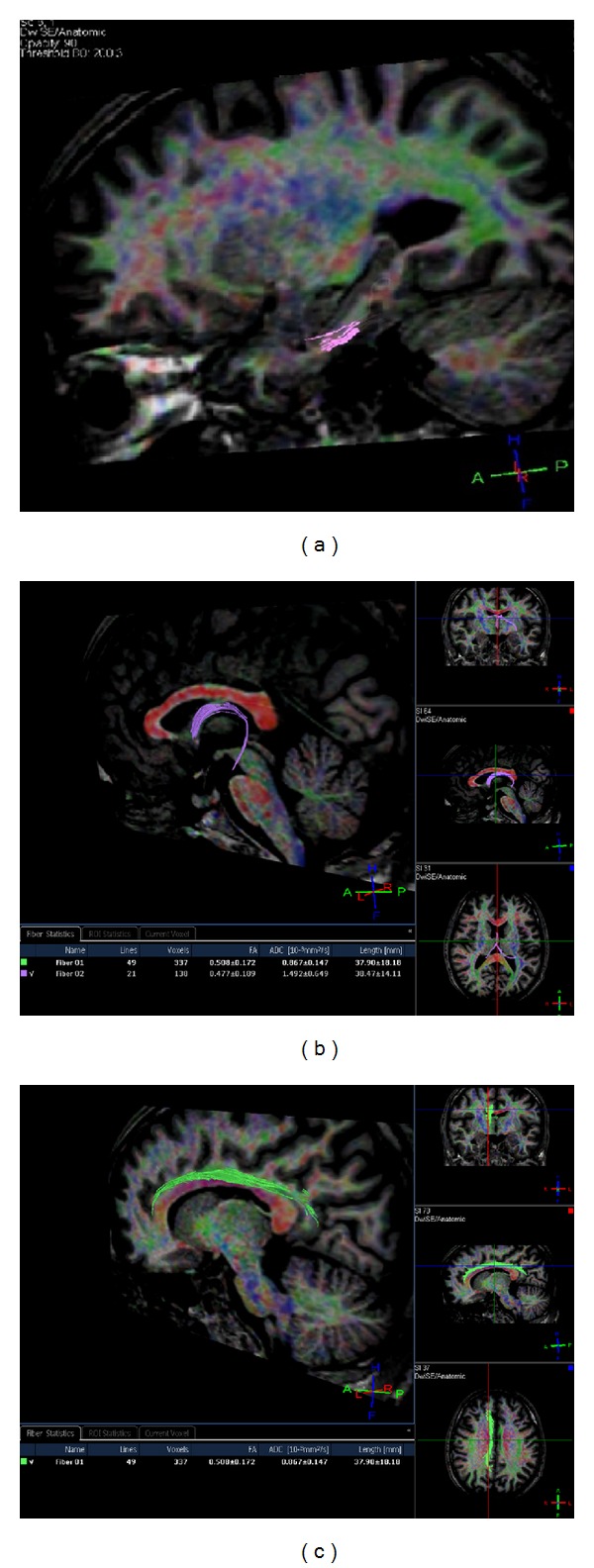
Fiber tracking was performed for 3D segmentation of the two major white matter tracts of the limbic system, the fornix (b), the cingulum (c), and additionally the parahippocampal gyrus (a).

DTI data were acquired using a single-shot spin-echo echo planar image (SE-EPI) sequence. The diffusion sensitizing gradients were applied simultaneously along sixteen noncollinear directions (*b* = 1000 s/mm²) as well as an acquisition without diffusion weighting (*b* = 0). The other acquisition parameters were TR = 8108 ms, TE = 75 ms, NSA = 3, flip angle = 90°, FOV = 224 mm, matrix = 256 × 256 pixels, 2 mm axial slices, and no slice gap. Three averages were applied for sufficient signal-to-noise ratio (SNR). Eddy current artifacts were minimized by the intrasequence registration tool utilized in the postprocessing. Fiber tracking was performed for 3D segmentation of fornix and cingulum as two major white matter tracts of the limbic system, and additionally for the parahippocampal gyrus (Figure 1). The fiber assignment by continuous tracking (FACT) algorithm, which starts tracking by every single voxel and goes over all the voxels of the image volume, was used for reconstruction of these fiber tracts [21]. The fiber tracking was performed with manually defined region of interest (ROI) placed according to color-coded maps based on the guidelines of Concha et al. [12]. Fiber tracking was terminated with a fractional anisotropy (FA) threshold of 0.20 and when the angle between two principal Eigen vectors was greater than 70°. Quantitative analysis was obtained from the statistical evaluation of parameters of pixels occupied by the reconstructed fibers. The diffusivity parameters FA and apparent diffusion coefficient (ADC) measurements were calculated for each selected fiber bundle and also for the hippocampus, amygdala, and parahippocampal gyrus as the gray matter components.

### 2.3. Statistical Analysis

Data were analyzed using statistical software (SPSS, version 16). The level of significance was set at *P* < 0.05 for all tests. We used the Kolmogorov-Smirnov test to verify the normal distribution of all variant groups. Kruskal-Wallis test was used to examine alterations of the parameters of the cases changed by age. The Mann-Whitney *U* test was performed after the Bonferroni correction to determine the difference between groups. Cross-table statistics were used to compare categorical variables (K-square, Fisher). For determining the statistically significant relationship between parameters Spearman correlation analysis was performed.

### 2.4. Results

**Figure 2:**
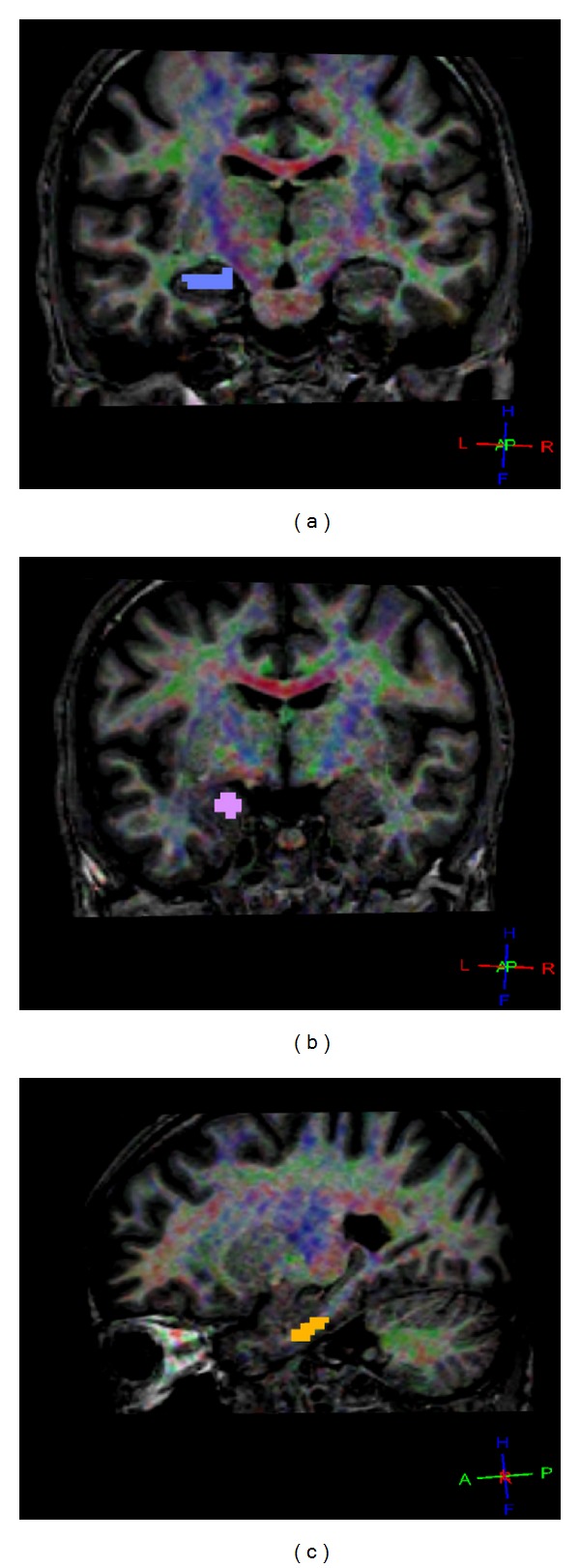
Examples of ROI placements on the hippocampus (a), the amygdala (b), and the parahippocampus (c) obtained from a 33-year-old man.

3D reconstructions were performed of the fiber structures of the fornix and the cingulum and the parahippocampal gyrus for all 31 subjects. Examples of ROI placements on the hippocampus, the parahippocampus, and the amygdala obtained from a 33-year-old man are shown in Figure 2. Diffusivity parameters and fiber characteristics for the cingulum, the hippocampus, the parahippocampus, and the amygdala of the left and the right hemispheres were calculated and evaluated separately. Two-sided paired *t*-tests revealed no differences between the two hemispheres, neither for diffusivity parameters nor for fiber characteristics. The mean fiber and nonfiber FA and ADC values for the left and the right hemispheres were used for statistical evaluation.

According to the results of Spearman correlation analysis, a negative correlation was found between age and left hippocampal FA measurements (*r* = −0.375, *P* = 0.038). The right parahippocampal gyrus FA values showed a moderate negative correlation with age (*r* = −0.486, *P* = 0.006). There were also weak negative correlations between the left parahippocampal gyrus, the fornix FA values, and age (*P* < 0.05). Interestingly, the ADC values of the right amygdala and the left cingulum showed a decrease with aging while the ADC values of the left hippocampus increased with age (*r* = 0.387, *P* = 0.031), as expected. No other statistical relationship was found between the other parameters and age.

**Figure 3:**
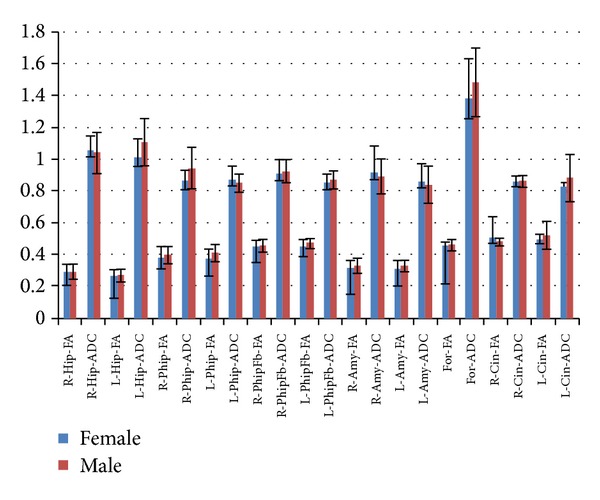
The ADC values of hippocampus, parahippocampus, and fornix were found to be higher in women.

Correlation related with sex showed a weak negative relationship between the left hippocampal, the right parahippocampal, and the left cingulum ADC values for men in comparison with women (*P* < 0.05). The ADC values of hippocampus, parahippocampus, and fornix were found to be higher in women (Figure 3).

**Figure 4:**
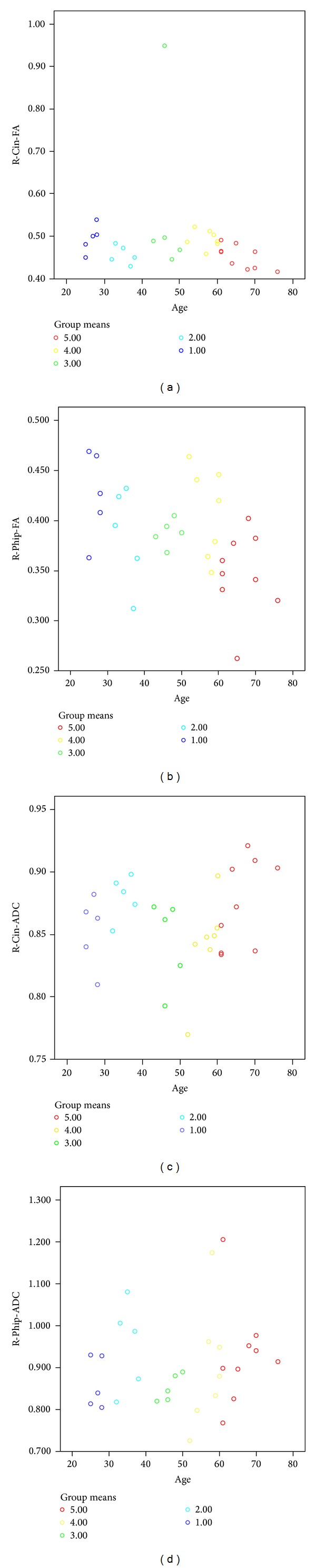
In group analysis, both sides parahippocampal and cingular FA and ADC values were statistically different between groups (*P* < 0.05).

In group analysis according to decades, both sides parahippocampal and cingular FA and ADC values were statistically different between groups (*P* < 0.05) that FA decreased and ADC increased with age (Figure 4).

In hippocampal and parahippocampal measurements, FA values showed negative correlation with ADC values (*r* = −0.386, *P* = 0.032) and 13 a positive correlation with each side. The correlation for the amygdala FA values was significant at *P* < 0.001, *r* = 0.662 and not significant for 14 the ADC values.

The fiber FA and ADC parameters of the right parahippocampus (*r* = −0.448, *P* = 0.011), fornix (*r* = −0.605, *P* ≤ 0.001), and right cingulum (*r* = −0.521, *P* = 0.003) demonstrated a negative correlation. The left cingular FA negatively correlated with the right cingular ADC. FA values of parahippocampal fibers for each side showed no correlation, while cingular fibers correlated significantly (*r* = 0.373, *P* = 0.039).

## 3. Discussion

DTI is rapidly becoming a widely available imaging technique with a myriad of applications. The possibility of discerning the orientation of white matter bundles and the ability to reconstruct their 3D structure in vivo has opened the door to selective studies of fiber tracts both in the healthy and diseased human brain. In healthy older subjects, anisotropy reduces and ADC increases in white matter with increasing age [22]. Pathological white matter axonal degeneration or demyelination shows reduced anisotropy and increased mean diffusivity, as, for example, in multiple sclerosis [23], amyotrophic lateral sclerosis [24], and Alzheimer's disease [25]. These changes may reflect demyelination, axonal loss, or edema [26, 27]. Generally, reduced FA in white matter suggests less coherence of fibers (e.g., crossing fibers) or less dense fibers. In contrast, increased ADC may suggest immaturity or degeneration in this region.

Several DTI studies, including neonates [28], children [29], and/or adolescents [30], have evaluated white matter changes of the brain in the normal aging process. Diffusion-tensor tractography has been used to delineate the fornix and/or cingulum in healthy volunteers [31–33], as well as patients with epilepsy and schizophrenia [12, 34]. Concha et al. [12] reported the diffusion characteristics of the fornix and cingulum with CSF suppression in healthy, young adults. Recently, Stadlbauer et al. [13] evaluated age-related changes of the fornix and cingulum with fiber tracking. Sullivan et al. [35] also mentioned them in a study of lateral and interhemispheric white matter fiber tracking in normal aging. However, to the best of our knowledge, no study has reported the diffusion characteristics of the whole components of the limbic system, including the hippocampus, amygdala, parahippocampal gyrus, cingulum, and fornix with DTI.

In the present study, we investigated the age-related changes of quantitative diffusivity parameters and fiber characteristics of the limbic system in healthy volunteers. Some of our FA values for the fornix and the cingulum showed a difference from the data reported in previous studies [12, 13, 35]. Concha et al. [12] and Stadlbauer et al. [13] found higher FA values while Sullivan et al. [35] found lower FA values in the fornix and cingulum. The ADC values were in agreement with their results. The observed discrepancies between studies may be due to clinical differences in the populations studied as well as methodological differences in anatomical definitions of these two bundles. Concha et al. interpolated data with eight averages in a scan time of more than 9 min. The interpolation may have reduced the SNR and the long scan time may have increased motion artifacts. Both the SNR and motion artifacts have a strong influence on the quality of DTI data and the outcome of fiber tracking [36].

In the current study, an age-related modest decline of FA in the fornix but not in the cingulate bundles was observed, as reported in previous studies [13, 35]. Zhang et al. also observed no FA changes of the cingulum in elderly controls while there were reductions especially in the left posterior cingulate region in mild cognitive impairment patients [37]. Furthermore, significant relative changes per decade of age were found in cingulum and parahippocampal gyrus FA measurements. In a retrospective view, the difference was thought to originate from the results of elderly patients in the fifth group.

Looking at the limbic system as a whole, the fornix includes fibers originating in the hippocampus and terminating in the mammillary bodies and septal nuclei, while the cingulated and parahippocampal gyri and septal cortex are connected via the cingulum. Left hippocampal FA values showed a decrease with normal aging as reported in volumetric studies [38, 39]. The left hippocampus is a participant in the recall of the spatial memories. When studying the hippocampal lesions in rats, Eichenbaum [40] and his team found that the left hippocampus is critical for effectively combining the “what,” “when,” and “where” qualities to compose the retrieved memory. This makes the left hippocampus a key component in the retrieval of spatial memory. However, Spreng and Mar [41] found that the left hippocampus is, in fact, a general concentrated region for binding together bits and pieces of memory composed not only by the hippocampus, but also by other areas of the brain to be recalled at a later time. This FA decrement in this region may be useful to explain the impairment of the memory functions in elderly people.

In this study the amygdala involved in signaling the cortex of motivationally significant stimuli such as those related to reward and fear in addition to social functions showed no significant differences with aging.

The cingulum that has autonomic functions regulating heart rate, blood pressure and cognitive, and attentional processing showed no difference, but the right parahippocampus that is connected by the cingulum and plays a role in the formation of spatial memory showed significantly lower FA values with aging. Yogarajah et al. found a parahippocampal FA decrease associated with poorer performance on material specific memory measures in temporal lobe epilepsy patients [42]. However, to the best of our knowledge these are the first results of parahippocampal gyrus tractography in normal aging.

Deterministic tractography methods require threshold values of FA to define each tract, although this practice may influence the resulting FA values calculated from the tract. For this study, the threshold was set to voxels with FA values greater than 0.20, to minimize inclusion of voxels with a high degree of partial volume contamination, and to avoid spurious tracts. The effect of choosing a threshold is unlikely to substantially affect the age-related FA and ADC changes with respect to the relative differences between structures. FA values derived from tractography are, in general, lower than FA values derived from ROI analysis due partly to this floor value, but primarily to variability of FA along the tracts themselves. ROI analysis defines structures on two-dimensional maps and generally includes the areas of higher FA in a particular tract. Tractography, however, includes a much larger portion of the tract, including the lower FA values near the ends, and obtains values that are lower overall. This variability in FA measures means that values obtained using identical methods can be compared, while it is difficult to compare absolute FA measures from different procedures.

Some limitations of this study must be acknowledged. First, the DTI acquisition was performed using a sixteen direction diffusion encoding scheme while the use of more directions could provide more robust estimates of anisotropy. Second, the diffusivity measures for the fornix were exceptionally high. Unique to the fornix is the fact that it is the only structure measured essentially surrounded by CSF, enhancing the possibility of exaggerating the influence of partial voluming (i.e., the inclusion of CSF rather than white matter in the voxel). Further, the observed fornix values may have been contaminated by CSF pulsation, again because of its location in the ventricles. In addition, we did not differentiate between cingulate bundles as the superior and the inferior extension.

## 4. Conclusions

The results of the current study showed that DTI in combination with quantitative fiber tracking is an accurate neuroradiology technique that provides information on age-related degeneration of the limbic system. We obtained significantly different changes in diffusivity parameters and fiber characteristics of limbic structures. Both ROI analysis and tractography revealed development trajectories for all limbic structures which, if compared to those in a patient population, could highlight the presence and timing of specific brain abnormalities associated with a particular disease.

## Figures and Tables

**Table 1:** Age and gender of the subjects.

Age	Number of subjects	Mean age ± SD	Gender FM/M
≥18, <30	5	22.1 ± 4.0	3/2
≥30, <40	5	32.5 ± 3.0	3/2
≥40, <50	7	45.0 ± 2.6	3/4
≥50, <60	7	53.8 ± 2.3	3/4
≥60, <70	7	65.3 ± 3.7	2/5
